# Is summer food intake a limiting factor for boreal browsers? Diet, temperature, and reproduction as drivers of consumption in female moose

**DOI:** 10.1371/journal.pone.0223617

**Published:** 2019-10-09

**Authors:** Rachel D. Shively, John A. Crouse, Dan P. Thompson, Perry S. Barboza

**Affiliations:** 1 Department of Wildlife and Fisheries Sciences, Texas A&M University, College Station, Texas, United States of America; 2 Alaska Department of Fish and Game, Division of Wildlife Conservation, Kenai Moose Research Center, Soldotna, Alaska, United States of America; Universidad Nacional Autonoma de Mexico Instituto de Investigaciones en Ecosistemas y Sustentabilidad, MEXICO

## Abstract

Food intake may limit the ability of browsing mammals to gain body mass during the growing season when the leaves and stems of woody plants are most abundant. Moose are highly productive browsers with high demands for energy and nutrients, particularly during lactation. Using an indigestible marker, we estimated dry matter intake of free ranging adult female moose with and without calves over three growing seasons. During the same period, we analyzed forage quality. Intakes were highest in late spring (280 ± 19 g·kg^-0.75^·d^-1^) when forage quality peaked; however, intakes declined by 39% throughout the summer as temperatures increased and as acid detergent fiber content of browse increased. Digestibility of dry matter declined over summer from 71% to 57% among browse. Intakes were similar for moose with and without calves. Heat loads may impair the ability of moose to consume sufficient energy and nutrients. Warming and habitat change can adversely affect browser populations when poor forage qualities and low dry matter intakes combine to suppress digestible intakes of energy and nutrients.

## Introduction

Mammals that consume the leaves and stems of woody plants consume a diet that is apparently abundant but often highly toxic or low in available nutrients and energy. The limits to maximum food intake of a browser determines the lowest quality the animal can tolerate to meet its demands for energy and nutrients [1] However, high demands for energy and nutrients further elevate intakes during periods of growth, post-winter recovery of energy and nitrogen (N) stores, and lactation. High intakes may also increase the costs of thermoregulation in hot environments [2]. In highly seasonal environments, forages change from low abundance (i.e. low biomass) and high quality (i.e. high concentrations of digestible energy and N) at the start of the growing season to high abundance and low quality at the end of the season [3–4]. Consequently, herbivores at northern latitudes are limited by low food availability in spring and their ability to sustain high intakes of low-quality forage during late summer and autumn. Annual variation in temperatures change the onset and duration of plant growth and thus the windows of nutrient availabilities for herbivores in seasonal environments [5–6].

The Family Cervidae is typically associated with strongly seasonal environments. Cervid habitats in shrub and forest provide a wide diversity of foods that include mushrooms, lichens, fruits, and the stems and leaves of forbs, graminoids and woody plants [7–10]. Although Cervids prefer foods that are high in N and low in fiber, they are able to consume plants with high concentrations of plant secondary metabolites that can reduce food intake and digestive efficiency [11–12]. Moose (*Alces alces*) are the largest Cervid and are also highly fecund with the ability to produce a litter of up to three offspring [13]. High demands are thus likely to drive food intakes of moose that consume stems of dormant browse low in available N and energy in winter [14]. Energy and nutrient gains may be limited by the low availability of forage in spring until plant growth is sufficient to support food intakes that are commensurate with both their large size and the high demands of supporting their offspring [15]. Warming conditions have been associated with declining productivity of moose populations [2,16–17]. While it is unknown how warming is affecting food intakes in moose, increasing summer temperatures can suppress food intakes of other mammals including those of domestic ruminants during lactation [18–19].

The abundance of forage at the end of winter is related to production of moose, that is the rate of producing twin calves declines as the rate of browsing on twigs increases [20–21]. Furthermore, ranges with higher quality of summer forages support greater densities of calves as well as cows and calves with greater body mass at the end of summer [22–24]. However, estimates of food intakes during summer have been limited to male moose and non-reproductive females because of challenges associated with observing cows with calves [25–26]. Estimates for lactating females are expected to be much greater [27–28].

Summer forage quality is related not only to the concentration of nutrients and gross energy but also to the rate at which fibrous plant cell walls and the contents of plant cells can be digested by the animal [1]. Although animals can eat more to offset the decline in concentration of nutrients and energy in the forage, food intakes are ultimately constrained by the ability to hold the slowly digesting fibrous plant cell walls of the lowest quality forages. Consequently, the ability to sustain high food intakes is directly related to the tolerance of low concentrations of digestible energy and nutrients in the diets of moose and other Cervids [29].

We studied food intake in relation to diet quality and environmental conditions for female moose during the summer when demands for mass gain and lactation are highest. We estimated the intakes of free-ranging adult female moose with and without calves during the growing season by measuring the concentration of an indigestible dietary marker in the feces. We used daily doses of the marker chromic oxide to estimate intake because alternative approaches such as 24 h observations of bite counts were not feasible especially for females with calves. We hypothesized that intakes of moose would be increased by lactation and by declining forage quality. We examined the effect of daily ambient air temperature on food intake to test the hypothesis that intakes would decline with increasing heat loads. We assessed the effect of seasonal changes in diet quality on digestible intakes of energy and N in moose with two approaches. Firstly, we measured the nutrient composition of five species of plants consumed by moose in our study area. Secondly, we measured fecal concentrations of total N and total phenols, which were compared with digestible energy and digestible N intakes of moose to assess their utility as indices of diet quality.

## Materials and methods

### Environment

The study was conducted at the Kenai Moose Research Center (MRC) located on the Kenai Peninsula, Alaska, USA (60°N, 150°W) from May through August (ordinal day 140–240) of 2014–2016. Temperature and precipitation were used to examine the effect of daily environment on food intake and forage quality. We collected air and soil temperature (5 cm depth) along with precipitation data from a National Oceanic and Atmospheric Administration (NOAA) U.S. Climate Reference Network weather station on site [30] (AK Kenai 29 ENE).

The MRC was established more than 50 years ago within the 1947 wildfire scar to include a mosaic of foraging habitats in various stages of succession for moose [31]. Animals and plants were studied within two outdoor enclosures (Pen 2 and Pen 3) of approximately 2.6 km^2^ (1.0 mile^2^) each. Current vegetation composition within each pen consisted of boreal forest (Paper birch (*Betula papyrifera*); Quaking Aspen (*Populus tremuloides*); White Spruce (*Picea glauca*); Black Spruce (*Picea mariana*)) in various successional states and non-forest patches. This patch variation is the result of ecological succession, browsing pressure by moose, and vegetation management activities. Vegetation management included mechanically crushing approximately 80 ha of 30-year-old forest within each pen in 2012 to improve foraging conditions of moose. Scouler willow (*Salix scouleriana*) is the most common willow and grows to tree size, but occurs at low plant densities. Pen 3 contains a 16 ha lake and many small ponds and bogs (≤ 0.2 ha) with standing water occuring within both pens.

### Forage

Forages were analyzed from the study site to measure forage quality and digestibility, which we later used to estimate food intake. Five species of plants were collected once a month within the sampling period including 4 browse species (Paper Birch; Quaking Aspen; Prickly Rose (*Rosa acicularis*); Scouler Willow) and one forb species (Fireweed; *Epilobium angustifolium*). Moose on this property have been observed frequently consuming the selected forage species. Each species was collected at 3 sites in each pen that had been mechanically crushed and where moose had been observed browsing. Plants were collected in both pens but Scouler willow was only collected in Pen 3 because it was so rare in Pen 2. At each location, a composite of 30 plants of each species were collected as 1 sample per species for the site. We collected forage samples by mimicking how moose browsed each plant species. We collected browse samples by removing leaves and clipping stems ≤ 2 mm diameter (*n* = 215). Early growth fireweed (<20cm tall) were pulled, rather than clipped, and this technique generally removed most of the fleshy underground stem along with the entire above ground portion of the plant. Older growth fireweed (>20 cm) was clipped 20 cm from the apex of the plant (*n* = 66). Samples were collected into plastic resealable bags then immediately frozen on dry ice before being transferred to a freezer and lyophilized in the lab (Freezone 18, Labconco, Kansas City, MO). We ground dried samples through a 1.0 mm mesh with a centrifugal mill (Retsch ZM 200, Hann, Germany).

Forages were analyzed for contents of dry matter, neutral detergent fiber, acid detergent fiber (ADF), and acid lignin [32] (Ankom Fiber Analyzer, Ankom Technology, Macedon, NY). We used an elemental analyzer (Flash EA1112, CE Elantech, Lakewood, NJ) to measure N content before and after extraction with acid detergent to estimate total and unavailable N. Available N was estimated as the difference between N in the whole sample and N in the post-ADF residue. We calculated digestible N content from available N content with the following relationships for browse (0.6629•available N– 0.1757) and forbs (1.03•available N) respectively [4].

We measured digestibility of dry matter using an in vitro method [33–34] (Daisy Incubator, Ankom Technology, Macedon, NY). Estimates of gross and digestible energy were from other forage studies in Alaska—forb and browse estimates were derived from those on the North Slope of Alaska [4] whereas graminoid estimates were derived from South-Central Alaska [35].

### Animals

The study conformed to ASM guidelines for the use of mammals in research [36]. All procedures for care, handling, and experimentation were approved by the Animal Care and Use Committee, Alaska Department of Fish and Game, Division of Wildlife Conservation (protocol # 0068–2018–48). All animals in this study were housed for use in further research.

We studied thirteen captive female moose (3–14 years old) for three summers (2014 *n* = 8; 2015 *n* = 12; 2016 *n* = 12). Females were not bred in 2015. Calves were born to 8 females in 2014 and 7 females in 2016; 13 sets of twins and 2 singleton births. Birthing occurred from ordinal day 127 to 155 (7 May– 4 June). Females lactated through the summer unless they lost calves to predators: 8 of 16 calves died in 2014 and 1 of 13 died in 2016, therefore 6 of 8 mothers in 2014 and 7 of 7 in 2016 mothers lactated through the end of the study period (S1 Table). Adult female moose were weighed in April before parturition and at the end of summer in September/October of each year (± 2 kg using a walk-on scale, MP Series Load Bars, Tru-Test Limited, Auckland, NZ). To monitor the condition of the animals, we measured maximum rump fat thickness (MAXFAT) [37] via ultrasonography (Ibex® Pro, E.I. Medical Imaging, Loveland, CO, USA) after immobilizing animals as described by Thompson et al. [38]. Ingesta-free body fat (IFBF) was calculated as IFBF = 5.61+2.05*MAXFAT [37]. Calves were weighed within 24h of birth (± 0.5 kg suspended in a nylon mesh sling, IN Series Linear Scale, Chatillon, NY; S1 Table). Postpartum maternal mass was estimated from the prepaturient mass in April minus the estimated mass of the conceptus (1.22• total offspring mass) [39].

In order to estimate intakes, we fed a known dose of indigestible marker and measured the concentration of that marker in fecal samples. We chose chromic oxide over other markers [1,40–41] because it was best accepted by moose and could be incorporated into a pelleted ration produced by the local mill. Continuous release devices were neither available nor feasible for multiple applications in moose. Although instantaneous estimates of intake rate can be made with bite counts, measures of daily food intake by bite counts were not feasible because direct observation of moose was only feasible for short periods. We therefore accustomed moose to consuming a daily dose of approximately 500 g marked pelleted ration to minimize repeatedly disturbing females, especially those accompanied by calves.

We used a complete pelleted ration (2.1% total N; 17.1% ADF; 81.2% dry matter digestibility) to administer the indigestible marker chromic oxide (Cr_2_O_3_) to moose at 0.22% of dry mass (Moose supplement #2; Alaska Pet and Garden, Anchorage AK). The dose of marked pelleted ration was measured as the difference in mass offered and refused each day. We collected and froze 30 g of marked pelleted ration each day to produce composite samples of the ration fed in each 2-week period. Fecal marker output was monitored from May to August. We collected fecal samples twice a week by following individuals until they were observed defecating. A sample of approximately 250 g wet mass was collected from the entire defecation and frozen on the day of collection. Doses and fecal collections were made in the morning to minimize the effect of diurnal variation on marker concentration [42–43]. Markers were dosed at 09:30 ± 1.7 h each day (*n* = 2829) whereas feces were sampled at 10:54 ± 2.3 h (*n* = 883) on the collection days.

Samples were dried to constant mass in either a forced air oven at 55°C or in a freeze-drier. Dried samples were ground individually through a 1.0 mm mesh in a centrifugal mill. Minerals were assayed in 6 replicates for marked pelleted ration samples and in duplicate for fecal samples. We combusted 0.25 g of ground sample in 10 mL of HNO_3_ (Fisher Scientific, Pittsburg, PA; 63.012 g/mole, ACS plus grade) by microwave digestion for 15 minutes at 210°C (OneTouch method for food; MARS 6 Microwave Digestion System, CEM Corporation, Matthews, NC). Digests were diluted with 60 mL deionized water (Millipore MQ -18MΩ). Chromium concentration was determined by microwave plasma atomic emission spectrometry at 427.480 nm (4200 MPAES, Agilent, Santa Clara, CA).

We measured fecal concentrations of phenols and N as indices of dietary antinutrients and nutrients respectively. We freeze-dried a subsample of every fecal sample for phenol analysis. We measured total phenolic activity in equivalents of Gallic Acid (GAE μg•mg^-1^) by colormetric reaction with Folin-Ciocalteu reagent in an adaptation of the Singleton method for a microplate reader (Spectramax Plus 384, Molecular Devices, San Jose, CA) [44]. Total fecal N was measured in one ground sample from each 2-week period (Flash EA1112, CE Elantech, Lakewood, NJ).

### Diet composition

Dried fecal samples were subsampled before grinding to create composite samples for diet analysis. Samples were pooled into 23 biweekly periods over all three years for each animal. Each pooled sample included approximately 5 g from each of 4 (± 2; n = 214) individual collections during the biweekly period from each animal. Fecal samples were analyzed for dietary components by microhistology at Washington State University (Wildlife Habitat Laboratory, Pullman, WA).

### Calculations

We used fecal concentrations of chromium from the indigestible marker to estimate dry matter intake. We calculated the daily chromium intake for each individual (ICr g·d^-1^) using the chromium concentrations in the consumed marked pelleted ration averaged over 5–day windows (S1 Method Validation) to accommodate daily variations in passage rate [45]. Total fecal output of dry matter (F g·d^-1^) was calculated as FCr ÷ ICr where FCr is the fecal concentration of chromium (g·g^-1^ DM). Microhistology results were corrected for digestibility to estimate diet composition (g component ·g diet^-1^) [46]. Browse and forb digestibility were estimated using the forage analysis results of this study. Graminoid digestibilities were derived from a previous study of moose forages in the Southcentral Alaska [35]. We estimated the overall digestibility of dry matter in the diet (Z g·g^-1^) as the weighted average of the component forages. Dry food intake (I g·d^-1^) was calculated from fecal output of dry matter (F g·d^-1^) and the dry matter digestibility of the diet (Z g·g^-1^) as F÷Z.

### Statistical analysis

All analyses were conducted in Stata 15.1 (StataCorp, College Station, TX). We report mean ± *SD*. Plant composition varies with growth and senescence as well as spatially due to environmental conditions so we used mixed-effects regression to analyze temporal and spatial variation of shrub and forb digestibility, available N and ADF for each plant species as well for the browse as a group. Fixed effects in the full model included pen, year and ordinal day (OD, OD^2^, OD^3^). To test if intake was varying with forage quality, we also used mixed-effects regression to analyze dry matter intake variation with forage quality. Fixed effects in the full model included pen, year, ADF and available N for browse and forb. We used the robust Huber/White sandwich estimator [47–48] to relax assumptions of normal distribution and homogeneity of variances for mixed-model regressions [49]. We compared model coefficients with zero using a *z* test and examined fixed effects with post hoc Wald tests, both at *P* < 0.05. Fixed effects were sequentially removed from the model when coefficients and post hoc tests were not significantly different from zero.

Three animals were dropped from intake calculations due to inconsistent consumption of the marked pelleted ration. We ran the package BACON in STATA to test for outliers [50] (S4 Table). Intake results were also censored if the estimate of dry matter consumed exceeded 10% of body mass (n = 217; S4 Table). We therefore removed intake estimates that would correspond to a gut capacity far above the general limit of 25% body mass in herbivores [1]. We used mixed effects regression to analyze variation in intake (total intake, intake by forage group, digestible energy intake, total N intake, available N intake) and fecal phenol concentration. Models included individual animal as a random effect to account for repeated measures. We tested the collinearity of OD and daily mean air temperature using variance inflation factor (VIF) score and both were below the threshold of 4 that would indicate significant collinearity (OD 1.35 and temp 1.38) [51]. Fixed effects in the full model included reproductive status, pen, daily mean air temperature, year, OD, and OD^2^ with digestible N and energy intakes included for fecal phenol concentration. Test of coefficients and model reduction were as described above. Data is available in the supplementary files (S1, S2, S3, and S4 Tables).

## Results

### Environment

**Fig 1:**
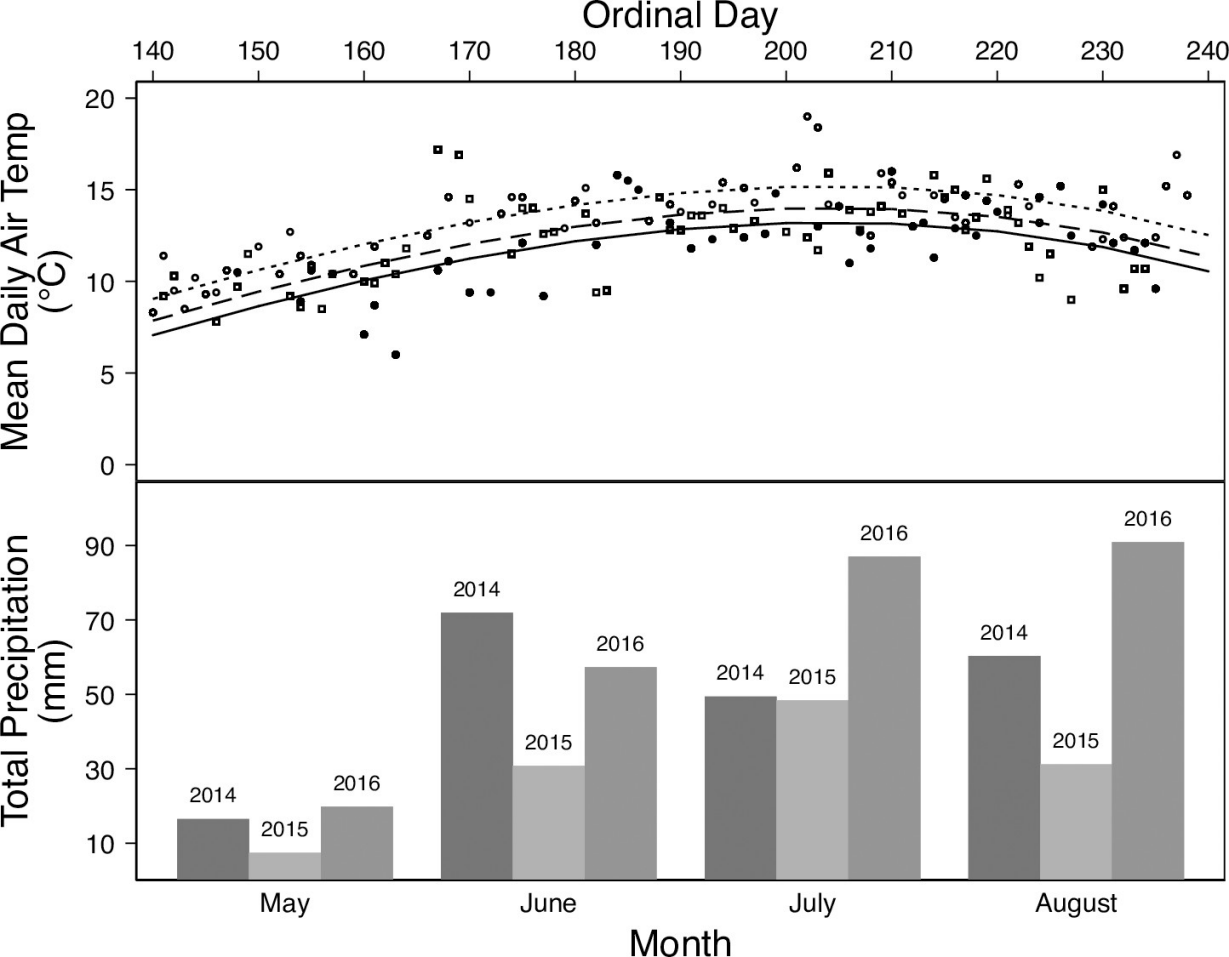
Environmental conditions. Mean daily air temperature (°C; top panel) and total monthly precipitation (mm; bottom panel) from May to August (ordinal day 140–240) of 2014–2016 recorded by NOAA U.S. Climate Reference Network weather station (AK Kenai 29 ENE) at the Kenai Moose Research Center, Kenai Peninsula, Alaska, USA. Symbols are observed data. Lines are predicted relationships between temperature and time for each year. Key: 2014 –solid line and solid circle; 2015 –dashed line and hollow circle; 2016 –dash-dotted line and hollow square. The period of early lactation is from ordinal day 140 to 170.

Temperatures of the soil and air define the window for plant growth. Daily average soil temperatures were above freezing during the study period (3.2 to 15°C). Similarly, mean daily air temperature was above freezing throughout the season with the highest temperatures recorded in 2016 (Fig 1). Total precipitation was also highest in 2016 (255.0 mm) and lowest in 2015 (117.8 mm; Fig 1).

**Fig 2:**
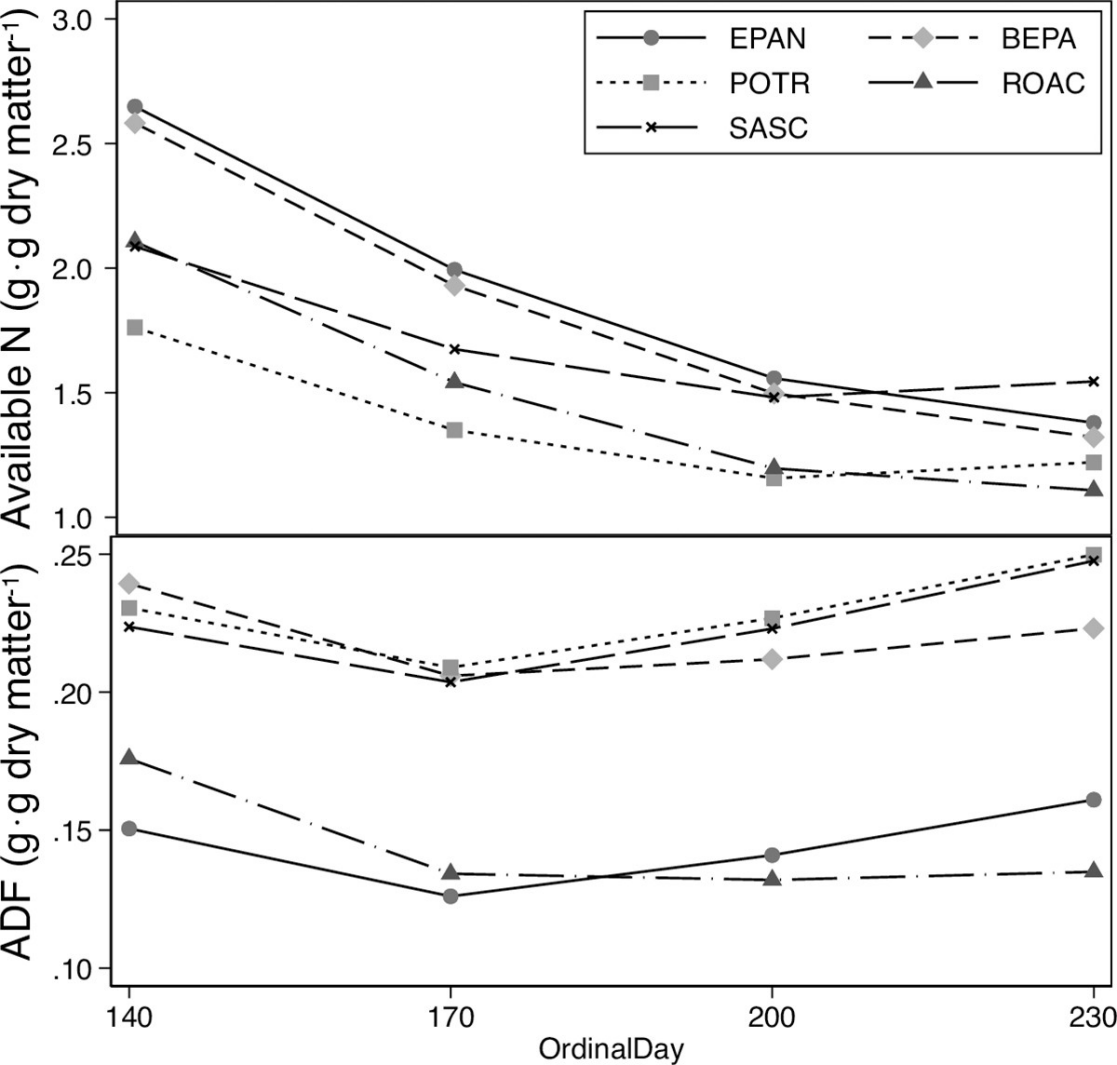
Forage quality. Phenology of available N (g·100 g^-1^; top panel) and fiber content (ADF g·g^-1^ dry matter; bottom panel) of forages available to moose through the summer (May–August; ordinal day 140–240) at the Kenai Moose Research Center, Kenai Peninsula, Alaska, USA, in 2014–2016. Symbols are estimated margins for each species. Lines are predicted relationships from mixed model regressions of concentration against time. Key: *Betula papyrifera* (BEPA)–dashed line and gray diamond; *Populus tremuloides* (POTR)–dotted line and gray square; *Rosa aciculari* (ROAC)–dashed line with dots and black triangle; *Salix scouleriana* (SASC)–short dashed line and black x’s; *Epilobium angustifolium* (EPAN)–solid line and black circle. The period of early lactation is from ordinal day 140 to 170.

Forage digestibility and available N content decreased while fiber content (ADF) increased through the summer (Fig 2). Fixed effects in the final model for forage quality included year and ordinal day (OD^2^ and OD^3^) for N content and year and ordinal day (OD^2^) for ADF content (S3A Table). Consequently, forage N declined with increasing fiber content. Digestibility of dry matter declined over summer amongst browse species (71% to 57%) and in the forb (83% to 72%).

### Animals & diet composition

Females weighed 313–472 kg in spring. Pregnant females delivered 24.8 (± 6.3) kg of neonatal mass, which provided an estimated postnatal maternal mass of 392 (± 29) kg. Females gained mass over the summer to attain autumn body weights of 413–572 kg. Maximum fat thickness on the rump increased from spring (0.3–1.7 cm) to autumn (2.1–7.8 cm) with a corresponding increase in body fat content from 7 (± 1) % in April to 15 (± 3) % in autumn. The average body mass of mothers was 444 (± 27) kg whereas non-reproductive females were 476 (± 30) kg in autumn (S1 Table).

Browse, particularly *Salix* and *Betula* species, were the largest component of the diet of moose (*X =* 57.8% (± 17.7); Range = 14.1–94.0%). The diet included a variable fraction of forbs (*X* = 33.6% (± 18.3); Range = 0–78.3%) usually with lesser amounts of graminoids (*X* = 8.5% (± 14.0); Range = 0–76.8%) and other items (e.g. ferns, berries, sedges, moss) (S2 Table).

### Intakes

**Fig 3:**
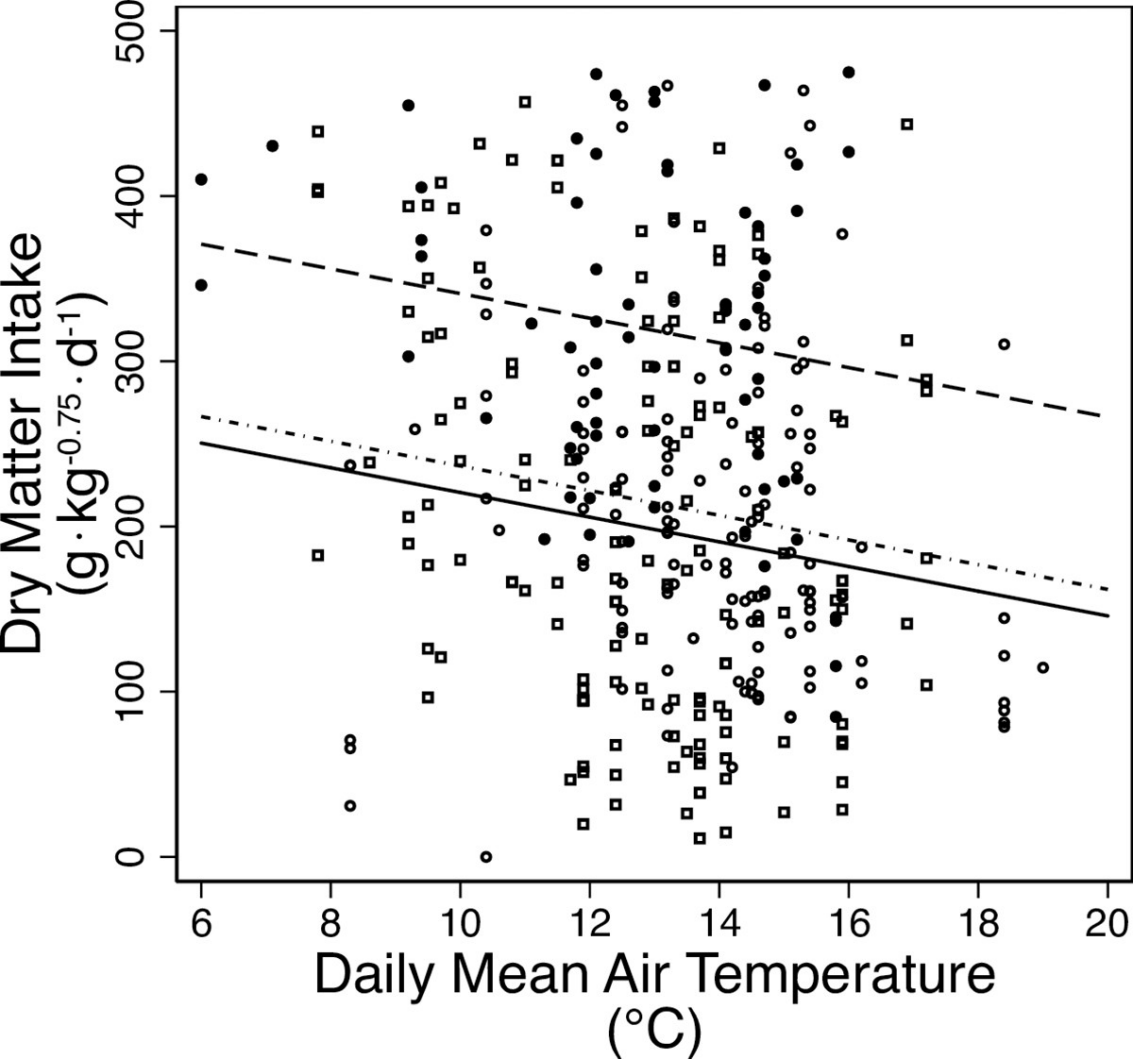
Intakes. Estimated daily dry matter intake (g•kg^-0.75^•d^-1^) of female moose from indigestible marker dosed in summer (May–August; ordinal day 140–240) at the Kenai Moose Research Center, Kenai Peninsula, Alaska, USA in 2014–2016. Symbols are observed data. Lines are predicted relationships from mixed model regressions of intake against mean daily air temperature (°C) for each year. Key: 2014 –solid line and solid circle; 2015 –dashed line and hollow circle; 2016 –dash-dotted line and hollow square.

Mean intake of dry matter was equivalent to 5.0% of body mass (±2.6%). Food intake decreased by 39% over the season in each year as air temperatures increased from a daily mean temperature of 6°C (280 ± 19 g·kg^-0.75^·d^-1^) to 19°C (176 ± 22 g·kg^-0.75^·d^-1^; Fig 3). Browse intake decreased while forb intake increased through the season (Table 1). Graminoid intake was highest at the beginning of the season but decreased quickly thereafter (Table 1). Intake was the same for reproductive and nonreproductive females. Fixed effects in the final models for total DM intake, shrub intake, forb intake, and graminoid intake included year, daily mean air temperature, and OD (Table 2).

**Table 1:** Intakes.

Component	Birth	Peak lactation	Late lactation	End of season
Browse	15.1 (± 1.4)	13.7 (± 1.2)	12.3 (± 1.1)	10.9 (± 1.1)
Forb	4.4 (± 0.9)	5.9 (± 0.6)	7.5 (± 0.6)	9.0 (± 0.9)
Graminoid	12.2 (± 2.4)	3.5 (± 0.6)	0.0 (± 0.3)	1.4 (± 0.5)
Total intake	24.8 (± 1.1)	23.0 (± 1.0)	21.2 (± 1.3)	19.4 (± 1.7)
Body Mass (kg)	414 (± 34)	431 (± 29)	446 (± 28)	463 (± 31)

Estimated daily dry matter intakes (kg•d^-1^ ± SD) and total body mass (kg) of female moose in summer (May–August; ordinal day 140–240) at the Kenai Moose Research Center, Kenai Peninsula, Alaska, USA in 2014–2016. Diets were determined by microhistology and corrected for digestibility. Intakes are linear estimates from the mixed model regression against time at birth (OD 140), peak lactation (OD 170), late lactation (OD 200) and end of season (OD 230) for the total diet and its principal components. Body mass was interpolated between spring and autumn. Maternal mass in spring was corrected for the estimated mass of the conceptus, which was derived from neonatal mass.

**Table 2:** Models.

		Dependent Variable (Y)		
Parameters and main effects	Level	Dry matter intake	Digestible energy intake	Digestible N intake
Observations		348	348	348
χ^2^ [df]		39.99 [4]	51.95 [4]	491.59 [4]
*P*		<0.0001	<0.0001	<0.0001
Intercept		592.3635	8.6361	36.0956
Reproductive	Non-Pregnant	base	base	base
	Pregnant	**—**	—	—
Pen	2	base	base	base
	3	**—**	**—**	**—**
Day	Ordinal Day	-0.9087	-0.0155	-0.3012
	Ordinal Day^2^	**—**	**—**	-0.0007
Year	2014	base	base	base
	2015	-120.4057	-1.6260	-1.8991
	2016	-104.3403	-1.5578	-2.7644
Air Temperature	Daily Mean	-7.4723	-0.1058	—

Fixed effects of mixed model regressions for repeated measures of daily intakes of dry matter (g•kg^-0.75^•d^-1^), digestible N (g•kg^-0.75^•d^-1^), and digestible energy (MJ•kg^-0.75^•d^-1^) of female moose in summer (May–August; ordinal day 140–240) at the Kenai Moose Research Center, Kenai Peninsula, Alaska, USA in 2014–2016. The full model of main effects and interactions (X) was reduced by sequentially removing non-significant effects (—; *P* > 0.05 for χ^2^ statistic).

**Fig 4:**
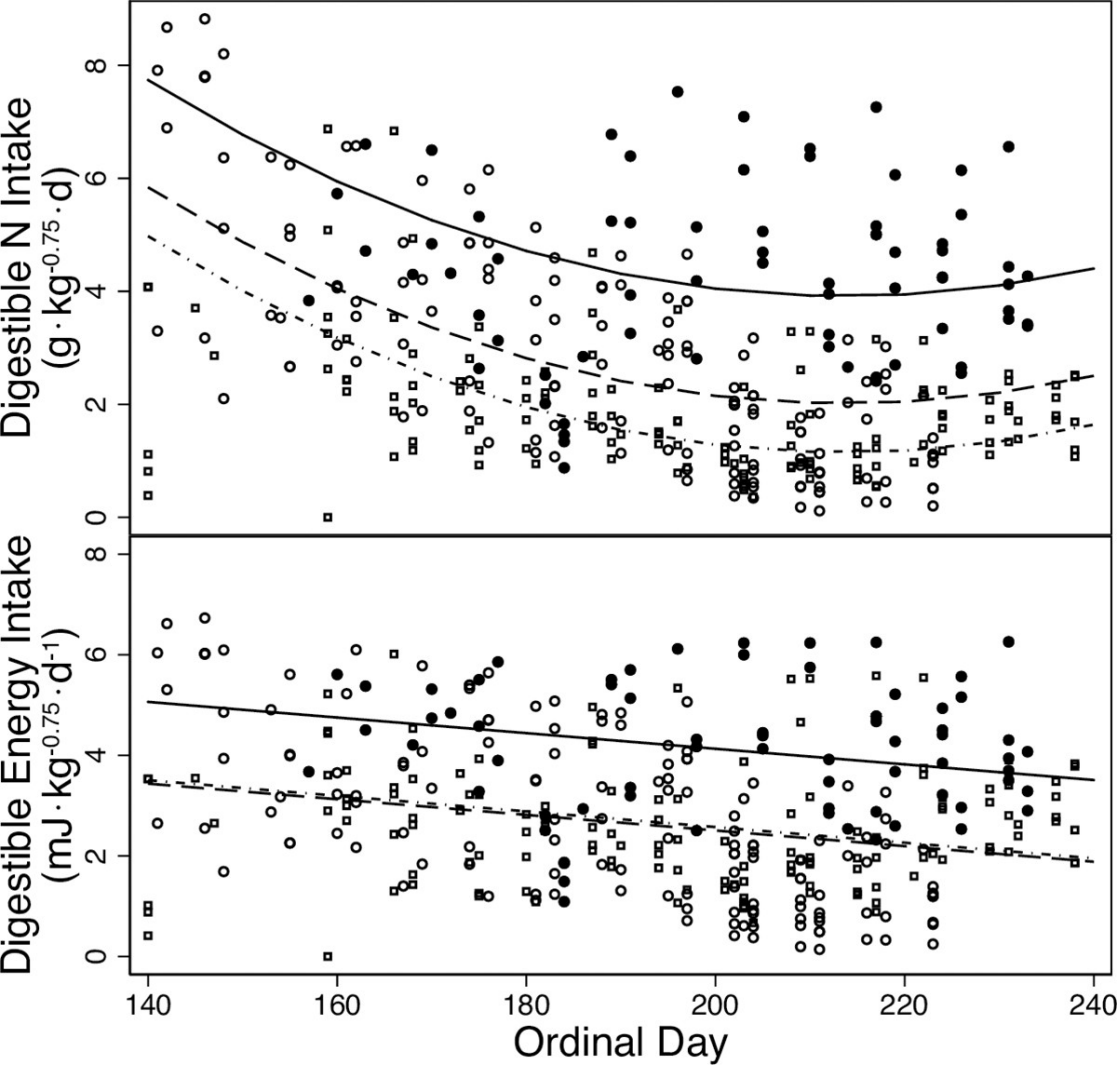
N and energy intakes. Daily intakes of digestible N (g•kg^-0.75^•d^-1^; top panel) and digestible energy (MJ•kg^-0.75^•d^-1^; top panel) of female moose in summer (May–August; ordinal day 140–240) at the Kenai Moose Research Center, Kenai Peninsula, Alaska, USA in 2014–2016. Symbols are observed data. Lines are predicted relationships from mixed model regressions of intake against time for each year. Key: 2014 –solid line and solid circle; 2015 –dashed line and hollow circle; 2016 –dash-dotted line and hollow square. The period of early lactation is from ordinal day 140 to 170.

The nutritional content of the diet varied with time and temperature. Digestible energy intake decreased over the season (Fig 4) from OD 140 (3.7 ± 0.2 MJ·kg^-0.75^·d^-1^) to OD 230 (2.4 ± 0.2 MJ·kg^-0.75^·d^-1^). Fixed effects in the final models for digestible energy intake included year, daily mean air temperature, and OD (Table 2). Increasing temperatures reduced digestible energy intake from 3.7 ± 0.3 to 2.2 ± 0.3 MJ·kg^-0.75^ d^-1^ between 6°C and 20°C. Digestible N intake decreased over the season but began to increase at the end of the season from OD 140 (5.9 ± 0.7 g· kg^-0.75^·d^-1^) to OD 230 (2.2 ± 0.1 g· kg^-0.75^ d^-1^). Fixed effects in the final models for digestible N intake included year OD and OD^2^ (Table 2). Digestible energy and N intakes varied by year (Fig 4); intakes were greatest in 2014 for both digestible energy (*X* = 4.1 ± 1.3 MJ·kg^-0.75^·d^-1^) and digestible N (*X* = 4.3 ± 1.5 g· kg^-0.75^·d^-1^; Fig 4). When ordinal day and temperature were excluded from the mixed model, dry matter intake increased with browse quality (decreased ADF and increased available N) but not forb quality (S3B Table).

### Fecal analysis and diet quality

**Fig 5:**
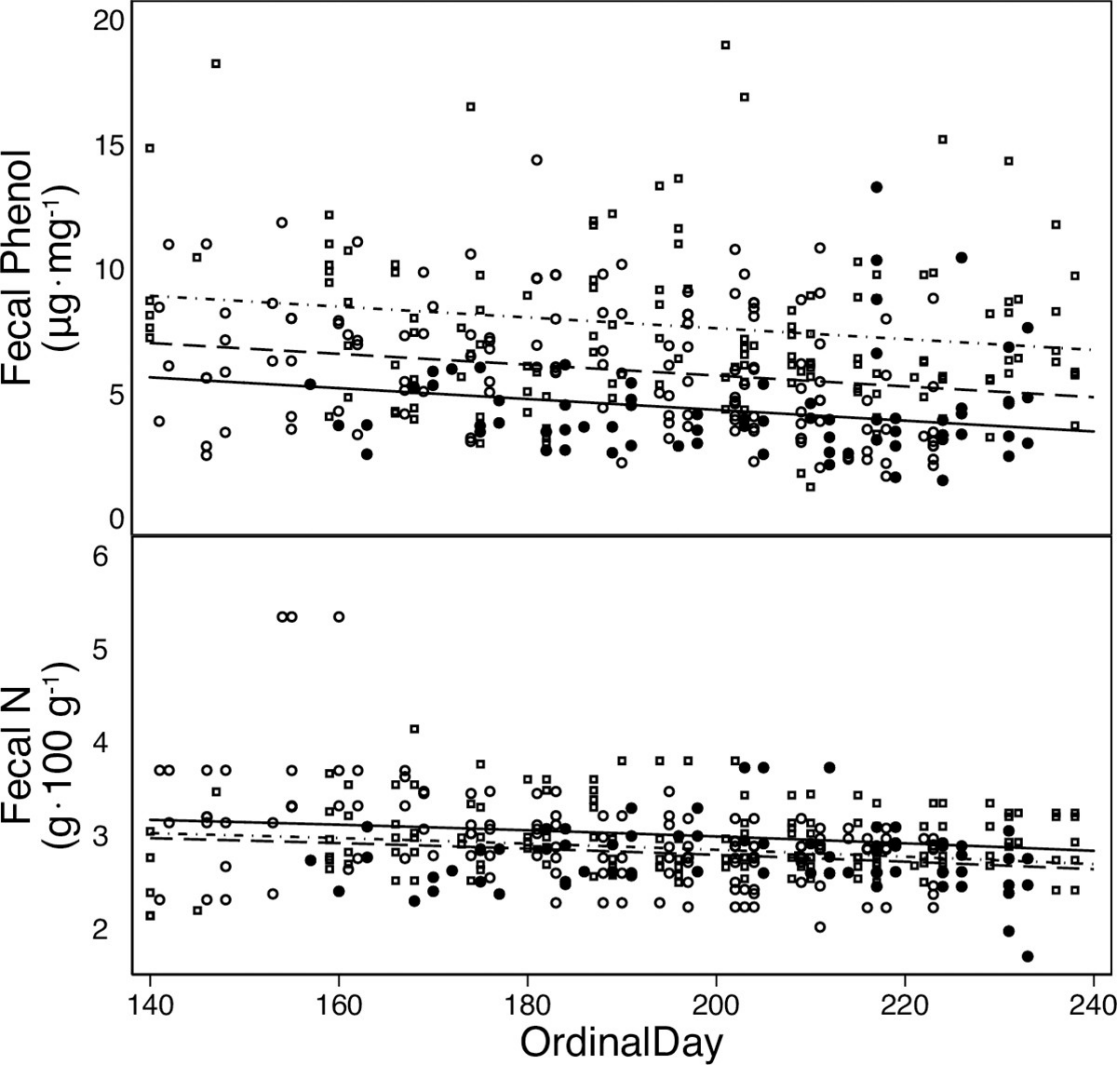
Fecal phenols. Fecal concentrations of phenol (GAE μg·g^-1^; top panel) and N (g·100 g^-1^) of female moose in summer (May–August; ordinal day 140–240) at the Kenai Moose Research Center, Kenai Peninsula, Alaska, USA in 2014–2016. Symbols are observed data. Lines are predicted from mixed model regressions of fecal concentrations against time for each year. Key: 2014 –solid line and solid circle; 2015 –dashed line and hollow circle; 2016 –dash-dotted line and hollow square. The period of early lactation is from ordinal day 140 to 170.

Fecal indices of diet quality varied during the season (Fig 5). Fecal phenol concentrations ranged from 1.2 to 18.9 GAE μg·g^-1^ and decreased over the season from 7.5 ± 0.5 to 5.5 ± 0.4 GAE μg·g^-1^ between OD 140 and 230 (Fig 5). Fecal phenol concentrations were not related to digestible N or energy (*P* = 0.789 and *P* = 0.486 respectively; S3C Table). Total fecal N also decreased over the season with available N intake. The highest annual concentration of fecal N (3.0 ± 0.1 g·100 g^-1^) was in 2014, which coincided with the lowest concentration of fecal phenol (4.4 ± 0.4 GAE μg·g^-1^). Total fecal N (g·100 g^-1^) was positively related to digestible energy intake (χ^2^ = 67.41, 5 df, *P* < 0.001) and digestible N intake (χ^2^ = 220.41, 5 df, *P* < 0.001).

## Discussion

We hypothesized that food intakes of adult female moose would increase over summer to offset the decline in forage quality. As predicted, forage quality measured as digestible energy and digestible N content declined over the growing season; however, moose intakes also declined, contrary to our hypothesis. We hypothesized that the demands of lactation would increase food intakes; however, lactating and nonreproductive females had similar intakes. Declines in dry matter intake coincided with increasing temperatures, which supported our hypothesis that food intake would be negatively affected by heat loads in summer. Fecal phenols were not related to digestible intakes of either energy or N as hypothesized. Conversely, our hypothesized relationships between fecal concentration of N and digestible intakes of energy and N were supported: fecal N was positively related to digestible intakes of energy and N.

### Plant context

In this study, browse was the largest component of the diet through most of the summer, which is consistent with wild moose populations throughout western North America [35,52–53] (S2 Table). A previous study at this site during a post burn period, birch leaves (*B*. *papyrifera*) were 56% of the summer diet (number of bites) with the remaining diet being mostly forbs (25%); grasses, sedges and aquatics (10%); and a small amount of willow (5%) [54]. During spring (late April-May), moose were observed eating a large amount of lichen (*Peltigera* spp.; 50% of diet) in this area [54], which we detected in fecal samples in early May of 2015 (S2 Table). Lichen may have been the most palatable forage available to moose at the end of winter before grass and forbs emerge in sufficient abundance. Similarly, the high intake of grass early in the season (49% of intake) indicates moose forage heavily on high quality, early season, non-browse species as they become available (Table 1).

Although forage quality decreased through the season (Fig 2), food intakes did not rise to offset the declines in digestible energy and digestible N content. Fecal N concentration declined with digestible N and energy content of the diet. However, variation in fecal N was small when compared with the large range of digestible intakes. In reindeer (*Rangifer tarandus*), endogenous N is 72–82% of total fecal N [55], that is dietary N contributes very little to fecal N. Consequently, fecal N is only a broad index of diet quality for moose.

Fecal phenols could reflect the dietary loads of toxins for moose because plant secondary metabolites in browse include high concentrations of polyphenolic compounds such as condensed tannins. However, fecal concentrations of phenol did not vary through the summer even though forages declined in digestible energy and digestible N content. Changes in the selection of forages by moose may attenuate loads of phenols. Although some plant secondary metabolites are heavily concentrated in new stems and leaves of *Betula* and *Populus* spp. in spring, condensed tannins increase towards senescence in the autumn [56–57]. Furthermore, some small phenolic compounds such as salicylates that are common in *Salix*, *Populus*, and *Betula*, are excreted in the urine [14,58]. Large phenolic compounds such as tannins are excreted in feces because salivary proteins bind linear condensed tannins [59–60]. Variation in fecal phenol concentrations among years were probably due to shifts in plant defenses that may be due to a combination of prior browsing by moose and other herbivores as well as growing conditions such as temperature and precipitation [61].

### Animal response

Estimated forage intakes of moose in this study were within estimated limits of intake based on bite rates from previous studies. Dry matter forage intakes of moose in this study were greater than those measured for moose fed formulated diets during the summer (5 ± 2.6% vs. 2.6–3.5% of body mass) [62]. Based on bite size and rate, the maximum intake rate for a 274 kg moose consuming leaves was observed at 26 g DM/min [63]. At that rate, a moose foraging for 10 hrs would have a daily intake of 5.7% body mass, which is within the observed daily foraging time of 9.9–10.5 hrs [26,64]. Moose may select specific foods in order to balance their N and energy requirements [65]. The mean digestible energy intakes we recorded for all moose during this study of 2.9 ± 1.6 MJ· kg^-0.75^·d^-1^ (Fig 4), was 4.8 times the estimated winter maintenance requirement and higher than the estimated energy demand for lactating moose (1.3–2.5 MJ· kg^-0.75^·d^-1^) [35,66–67]. These high energy intakes suggest that female moose were not constrained by energy supply in this habitat because females were able to gain body mass and thus increase energy stores through summer. Similarly, digestible intakes of N (*X* = 2.8 ± 1.9 g· kg^-0.75^·d^-1^; Fig 4) exceeded estimates for maintenance of body mass at (0.6 ± 0.1 N g· kg^-0.75^·d^-1^) [68]. However, at the lowest dry matter intakes in 2016, mean digestible N intakes of 1.8 ± 1.2 g· kg^-0.75^·d^-1^ were three times the maintenance requirement but only 1.4 times the estimated N demand for lactating moose (1.3 g· kg^-0.75^·d^-1^) [35]. Forage supplies of N may therefore constrain lactation or growth of calves in some summers especially when air temperatures are high [69].

Food intakes were equally high among females with and without calves, that is lactational demand did not elevate food intakes. High intakes of both lactating and dry moose would be consistent with natural selection for high appetites in a short summer season and with the high fecundity of moose, which relies upon accumulating body stores of energy and protein in summer for reproduction in the subsequent winter and spring [70].

High variance in our estimates of dry matter intake were probably due to variation in marker distribution and flow associated with changes in both food intake and diet composition through the season that affect passage of fluid, and different sizes of particulate digesta in moose [45,71]. Food intakes decreased by 22% as the diet of browse shifted from small amounts of emergent grass to forbs over the summer. We minimized diurnal artefacts of marker flow by dosing and collecting markers at the same time of day. We also used a running average of marker consumption over 5 days to best represent the daily dose rate and censored outliers of estimated intake.

### Summer limits

Moose populations may be adversely affected by the combined effects of warming and habitat change [19, 72–78]. Increasing air temperatures may lead moose to reduce time spent browsing while increasing the cost of thermoregulation [79–80]. Increasing spring temperatures in particular have a negative effect on moose densities, possibly due to increased stress in late spring prior to the shedding of winter coats [74]. Additionally, warming temperatures are predicted to increase insect herbivory, which could decrease moose browsing in winter [81]. Warmer spring temperatures also promote higher moose tick populations, which increases hair loss, weight loss, anaemia and secondary bacterial infections [82–86]. Moose flies and mosquitoes may harass moose to the extent that they move to different habitats and increase movement [87–88]. These combined constraints on moose related to summer environmental conditions could decrease moose production.

Warming temperatures will constrain productivity of populations through a combination of decreased forage quality and increased thermoregulatory costs. The warmest year of this study (2016) had the lowest dry matter intakes, the lowest digestible N and energy content of the diet, and the highest fecal phenol concentrations. This indicates that summer food intakes may become a limiting factor for these boreal browsers as temperatures increase. Warming and habitat change can adversely affect browser populations when poor forage qualities and low dry matter intakes combine to suppress digestible intakes of energy and nutrients.

## Supporting information

S1 Table Attributes of study animals. Body mass, birth date, number and fate of offspring.
(DOCX)

S2 Table Microhistology results. Microhistology results pooled by 2-week periods and averaged across all animals.
(DOCX)

S3 Table Forage quality, intake and fecal indices models. Fixed effects of mixed model regressions for repeated measures of **A.** shrub and forb digestibility (%), available N (g•100g^-1^), and ADF (g•g DM^-1^) of sampled forages, **B.** estimated daily dry matter intake when ordinal day and temperature were excluded (g•kg^-0.75^•d^-1^), and **C.** daily intakes of digestible N (g•kg^-0.75^•d^-1^) and digestible energy (MJ•kg^-0.75^•d^-1^) of female moose in relation to fecal concentration of N (g·100g^-1^) in summer (May–August; ordinal day 140–240) at the Kenai Moose Research Center, Kenai Peninsula, Alaska, USA in 2014–2016. The full model of main effects and interactions (X) was reduced by sequentially removing non-significant effects (—; *P* > 0.05 for χ^2^ statistic).
(DOCX)

S4 Table Method selection for censored results. We compared methods of removing outliers to limit the potential bias of censoring. We ran the package BACON in STATA to test for outliers with a p = 0.15 limit (Billor et al. 2000). The results of BACON on fecal output estimates present the number of outliers in the data prior to correcting for digestibility while the results of BACON on intake present the number of outliers after correcting for digestibility. We chose to censor data using a cutoff of 10% of body mass for intake results. Intake estimates over 10% of body mass would correspond to a gut capacity far above the general limit of 25% body mass in herbivores (Barboza et al. 2009). Censoring observations of intake >10% body mass reduced the mean intake by 54%. Censoring did not affect the distribution of samples over time even though the range of the number of observations per animal were reduced from 22–76 to 16–46.
(DOCX)

S1 Method Validation Model of chromium excretion. We devised a simulation model in the program STELLA (version 10.06 ISEE Systems, Lebanon NH) to examine the sensitivity of the estimation method to variation in the consumption of the marker ration and the quality of the diet. The model used published measures of food intake, food quality and digesta flow of moose (Clauss et al. 2011; Welch et al. 2015) to simulate pools of dry matter and marker in the rumen and the intestines (Fig A). The model predicted that marker concentrations in the feces would equilibrate after 5 days of dosing the marker at 15 mg·g^-1^ (Fig B, panel A) across a range of inputs for food intake (6400–15,400 g•d^-1^), and digestibility (0.93–0.54 g•g^-1^). Consequently, intakes estimated from marker concentrations in the model output were not significantly different from the simulated food intake averaged over 5 day intervals (Fig B, panel B).
We validated Cr as the indigestible marker chromic oxide (Cr_2_O_3_) in five female moose (body mass 270–306) on ad libitum browse in winter (February–March). Each animal was given 500 g of a supplement (0.8 g•g^-1^ digestibility) containing 636 ppm. The marker was not detected in the feces before dosing at -4 days. Marker concentrations increased on the day after each dose and declined within 2 days before the next dose was consumed. The marker disappeared from the feces within 5 days of consuming the last marker dose, which is consistent with the simulation model above (Fig C).
(DOCX)
