# Supplementary material for: Is summer food intake a limiting factor for boreal browsers? Diet, temperature, and reproduction as drivers of consumption in female moose
Source: PLoS One. 2019 Oct 9;14(10):e0223617. doi: 10.1371/journal.pone.0223617 (PMC6785127; doi:10.1371/journal.pone.0223617)
Supplement: S1 Table — Body mass, birth date, number and fate of offspring. (DOCX) [file pone.0223617.s001.docx]

|  | Year | Cayenne | Eleanor | Flo | Isabella | Jackie | Lily | Minnie | Olivia | Roxanne | Shiner | Sky | Stella | Wilma | Total |
| --- | --- | --- | --- | --- | --- | --- | --- | --- | --- | --- | --- | --- | --- | --- | --- |
| Cow Birth Year |  | 2012 | 2003 | 2012 | 2002 | 2003 | 2003 | 2008 | 2002 | 2009 | 2012 | 2012 | 2009 | 2012 |  |
| Pen ID | 2014 | 2 | 3 | 2 | 3 | 3 | 3 | 2 | 3 | 2 | 2 | 2 | 2 | 2 | 13 |
|  | 2015 | 3 | 3 | 3 | 3 | 3 | 3 | 2 | 3 | 2 | 2 | 3 | 2 | 2 | 13 |
|  | 2016 | 3 | • | 3 | 2 | 3 | 3 | 2 | 3 | 2 | 2 | 3 | 2 | 2 | 12 |
| Spring Mass (kg) | 2014 | • | 459 | • | 452.6 | 455 | 447 | 363 | 472 | 404 | • | • | 401 | • | 8 |
|  | 2015 | 326 | • | 313 | 364 | 386 | 408 | 345 | 386 | 329 | 331 | 327 | 374 | 321 | 12 |
|  | 2016 | 381.5 | • | 389 | 378 | 386 | 432 | 425.5 | 400 | 426 | 403 | 429 | 441 | 355 | 12 |
| Fall Mass (kg) | 2014 | • | 459 |  | 495 | 486 | 516 | 420 | 492 | 442 | • | • | 469 | • | 8 |
|  | 2015 | 413 | • | 426 | 522 | 459 | • | 457 | 572 | 466 | 447 | 442 | 494 | 438 | 11 |
|  | 2016 | 441 | • | 436 | 494 | 453 | 501 | 434.5 | 502 | 443 | 484.5 | 447 | 509 | 486 | 12 |
| Number of Calves | 2014 |  |  |  |  |  |  |  |  |  |  |  |  |  |  |
|  | Born | • | 2 | • | 2 | 2 | 2 | 2 | 2 | 2 | • | • | 2 | • | 16 |
|  | Survived | • | 1 | • | 2 | • | • | 1 | 1 | 1 | • | • | 2 | • | 8 |
|  | 2016 |  |  |  |  |  |  |  |  |  |  |  |  |  |  |
|  | Born | 2 | • | 2 | • | • | • | 2 | • | 2 | 2 | 2 | 1 | • | 13 |
|  | Survived | 1 | • | 2 | • | • | • | 2 | • | 2 | 2 | 2 | 1 | • | 12 |

S1 Table
