# Supplementary material for: Is summer food intake a limiting factor for boreal browsers? Diet, temperature, and reproduction as drivers of consumption in female moose
Source: PLoS One. 2019 Oct 9;14(10):e0223617. doi: 10.1371/journal.pone.0223617 (PMC6785127; doi:10.1371/journal.pone.0223617)
Supplement: S2 Table — Microhistology results pooled by 2-week periods and averaged across all animals. (DOCX) [file pone.0223617.s002.docx]

| Year | 2014 | | | | | | | 2015 | | | | | | | | 2016 | | | | | | | |
| --- | --- | --- | --- | --- | --- | --- | --- | --- | --- | --- | --- | --- | --- | --- | --- | --- | --- | --- | --- | --- | --- | --- | --- |
| Date Range | 5/24- 6/6 | 6/7-6/20 | 6/21- 7/4 | 7/5-7/18 | 7/19- 8/1 | 8/2-8/15 | 8/16-8/29 | 5/5-5/19 | 5/20- 6/2 | 6/ -6/16 | 6/17-6/30 | 7/1-7/14 | 7/15-7/28 | 7/29-8/11 | 8/12-8/25 | 5/10-5/24 | 5/25- 6/8 | 6/9-6/22 | 6/23- 7/6 | 7/7-7/20 | 7/21- 8/3 | 8/4-8/17 | 8/18-8/31 |
| Alnus viridis crispa leaf |  | 3.20 | 5.03 | 3.40 | 3.00 |  |  |  | 3.20 |  |  |  |  |  |  |  |  |  |  |  |  |  |  |
| Betula glandulosa papyrifera leaf | 7.12 | 6.90 | 12.56 | 4.87 | 8.90 | 8.65 | 6.80 |  |  | 8.13 | 13.53 | 15.55 | 15.03 | 15.18 | 10.69 | 3.88 | 12.62 | 8.64 | 8.27 | 12.22 | 16.20 | 13.91 | 9.13 |
| Betula glandulosa papyrifera stem | 11.88 | 10.14 | 12.95 |  |  |  | 6.30 | 20.65 | 9.07 | 5.70 | 10.30 | 14.07 | 15.40 |  | 16.90 | 19.35 | 7.18 | 3.43 | 5.40 | 12.80 | 2.10 | 7.80 | 13.15 |
| Cornus canadensis leaf |  | 5.03 | 5.75 | 6.00 | 15.40 | 22.40 | 4.80 |  |  | 4.00 | 3.95 |  |  | 6.75 | 7.90 | 18.30 | 8.00 | 11.37 | 14.60 | 7.70 | 4.70 | 10.00 | 5.20 |
| Ledum groenlandicum leaf |  |  |  |  |  |  | 3.10 | 6.17 | 10.70 |  |  |  |  |  |  | 3.70 |  |  |  |  |  |  |  |
| Ledum groenlandicum stem |  |  |  |  |  |  |  |  | 7.70 |  |  |  |  |  |  |  |  |  |  |  |  |  |  |
| Menziesia ferruginea leaf |  | 4.40 | 3.30 | 5.10 |  |  |  | 3.00 | 4.95 | 6.10 |  |  |  |  |  | 3.80 | 4.90 |  |  |  |  |  |  |
| Myrica gale leaf | 6.60 |  |  |  |  |  | 5.10 |  |  |  |  |  |  |  |  |  |  |  |  |  |  |  |  |
| Myrica gale stem | 10.50 |  |  |  |  |  |  |  |  |  |  |  |  |  |  |  |  |  |  |  |  |  |  |
| Oplopanax horridus stem |  | 11.60 |  |  |  |  | 4.50 |  |  |  | 3.50 |  | 4.60 |  |  | 3.20 | 3.90 |  |  |  |  |  |  |
| Populus balsamifera leaf | 7.85 | 2.30 |  |  |  | 3.70 | 6.93 |  | 4.00 | 8.40 | 6.00 | 8.05 |  | 7.70 |  |  |  |  |  |  |  |  |  |
| Populus tremuloides leaf | 5.07 | 19.70 | 7.60 | 13.30 | 6.70 | 8.54 | 8.24 |  | 9.53 | 7.38 | 7.64 | 6.67 | 9.63 | 10.01 | 15.17 | 7.30 | 6.58 | 14.46 | 12.01 | 10.60 | 13.93 | 16.30 | 13.35 |
| Populus spp stem | 5.83 | 8.95 | 10.92 | 11.80 | 8.33 | 6.55 | 11.80 | 9.39 | 6.54 | 6.97 | 6.10 | 6.37 | 9.00 | 7.30 | 5.40 | 5.83 | 4.65 | 5.40 | 5.00 | 6.20 | 6.95 |  | 8.33 |
| Ribes stem |  |  |  |  |  |  |  | 4.50 |  |  |  |  | 6.70 |  |  |  |  |  |  |  |  |  |  |
| Rosa acicularis leaf | 4.10 | 6.10 |  |  | 4.30 | 6.65 | 4.70 | 5.08 | 3.87 | 3.20 | 5.20 | 6.23 | 3.35 | 9.78 | 7.48 | 5.77 | 5.97 | 9.27 | 7.54 | 10.91 | 9.30 | 9.80 | 8.80 |
| Rosa acicularis stem | 7.65 |  |  | 4.30 | 13.07 | 16.70 | 6.43 | 7.74 | 6.24 | 6.06 | 8.88 | 5.43 | 4.70 | 10.83 | 2.70 | 4.48 | 4.28 | 3.27 | 5.17 | 4.90 | 9.10 | 2.40 | 3.90 |
| Rubus spp leaf |  |  | 6.50 | 2.90 | 4.93 | 4.08 | 3.70 |  | 5.30 | 2.57 | 5.33 | 3.30 | 6.55 |  |  |  |  | 4.40 | 5.40 | 6.95 |  |  |  |
| Rubus spp stem | 9.40 | 4.60 |  | 3.83 | 4.40 | 7.27 | 4.25 | 14.80 |  | 4.47 |  | 3.88 | 4.05 | 5.15 | 3.05 | 5.43 |  | 6.20 | 6.65 | 5.23 | 3.40 |  |  |
| Salix leaf | 19.92 | 15.20 | 20.95 | 20.25 | 11.93 | 17.92 | 11.96 | 9.10 | 18.20 | 19.13 | 18.57 | 18.60 | 19.59 | 18.25 | 22.53 | 17.37 | 35.70 | 25.95 | 21.65 | 25.10 | 24.52 | 25.20 | 34.39 |
| Salix stem | 11.83 | 7.08 | 11.80 | 12.23 | 6.77 | 10.60 | 11.62 | 12.36 | 13.21 | 14.29 | 15.28 | 13.96 | 7.51 | 8.58 | 9.47 | 13.33 | 11.57 | 11.83 | 9.32 | 10.40 | 8.30 | 9.05 | 9.74 |
| Spiraea stevenii beauverdiana |  |  |  |  |  | 5.15 | 3.20 |  |  |  |  |  |  |  |  |  |  |  |  |  |  |  |  |
| AA | 5.50 |  |  |  |  |  |  | 4.00 |  |  | 6.00 |  |  |  |  |  |  |  |  |  |  |  |  |
| Vaccinium vitis idaea leaf | 8.60 | 10.20 | 3.30 |  |  |  | 3.00 | 19.19 | 6.33 |  |  |  |  |  | 5.30 | 13.83 | 14.03 | 6.30 | 9.53 |  |  | 7.80 |  |
| Vaccinium vitis idaea stem |  | 4.75 | 3.55 |  |  |  |  | 10.75 | 6.50 | 7.90 |  |  |  |  | 5.30 | 6.13 | 5.10 |  |  |  |  |  |  |
| Viburnum edule |  |  |  |  |  |  |  | 4.50 |  |  |  |  |  |  |  |  |  |  |  |  |  |  |  |
| Viburnum edule stem |  |  |  |  |  |  |  |  |  |  |  |  |  |  |  | 8.75 | 5.70 |  |  | 4.00 |  |  |  |
| Shrub above | 4.66 | 3.93 | 2.63 | 2.77 | 1.75 | 2.88 | 3.90 | 2.92 | 2.76 | 2.59 | 2.36 | 2.50 | 1.72 | 2.63 | 2.82 | 3.64 | 2.16 | 3.06 | 2.61 | 1.17 | 1.93 | 2.55 | 2.00 |
| Unknown Shrub leaf |  |  |  |  | 3.00 |  | 3.10 | 3.60 | 3.60 | 3.10 | 4.05 |  | 3.10 | 2.65 | 3.13 | 4.80 |  | 2.40 | 7.03 |  | 3.40 | 2.55 | 2.50 |
| Unknown Shrub stem | 2.00 | 1.50 |  |  | 3.95 |  | 5.10 | 2.73 | 5.20 | 2.20 |  |  | 4.05 | 1.20 |  | 2.20 | 2.45 | 2.30 | 2.10 |  |  |  |  |
| **Total Shrubs** | 62.49 | 52.15 | 63.23 | 48.30 | 45.00 | 50.34 | 49.51 | 68.79 | 57.89 | 65.89 | 65.44 | 70.10 | 56.18 | 63.83 | 70.31 | 73.33 | 77.13 | 68.46 | 71.04 | 73.44 | 72.58 | 77.78 | 76.78 |
| Berry |  | 0.77 |  |  |  |  |  |  |  |  |  |  |  |  |  |  |  |  |  |  |  |  |  |
| Thorn |  |  |  |  |  |  |  | 0.24 |  | 1.40 |  |  |  |  |  |  |  |  |  |  |  |  |  |
| Equisetum | 0.37 |  | 2.57 | 2.36 |  | 2.50 |  |  | 1.49 | 1.27 | 0.15 | 2.72 | 0.20 |  |  |  |  | 3.37 | 2.75 | 0.34 | 1.15 | 0.31 | 1.30 |
| Chamerion Epilobium angustifolium | 5.90 | 6.58 | 9.00 | 36.60 | 45.14 | 40.83 | 38.59 | 3.15 | 9.84 | 13.83 | 22.79 | 20.85 | 37.09 | 28.14 | 20.87 | 8.33 | 14.77 | 12.45 | 18.20 | 20.18 | 19.80 | 19.33 | 19.51 |
| Comarum Potentilla palustris |  |  |  |  |  |  |  |  |  |  | 7.70 |  | 7.10 |  |  |  |  |  |  |  |  |  |  |
| Menyanthes trifoliata |  |  | 4.50 | 4.90 | 4.30 |  |  |  |  |  |  |  |  |  |  | 5.37 |  |  |  |  |  |  |  |
| Streptopus amplexifolius |  | 9.70 |  |  |  |  | 4.05 |  |  | 8.25 | 6.20 | 3.30 | 5.30 |  |  | 4.10 |  |  |  |  |  |  |  |
| Polemonium acutiflorum |  |  |  |  |  |  |  |  |  |  |  |  |  |  |  |  |  | 8.80 |  |  |  |  |  |
| Forbs above | 2.00 |  |  | 2.75 |  |  |  |  | 2.60 |  |  | 1.50 |  |  |  |  | 1.70 | 2.20 |  |  |  |  |  |
| Unknown Forbs | 3.30 | 6.17 | 4.50 | 4.70 | 3.10 |  | 1.90 |  | 2.15 | 1.87 | 3.46 | 2.90 | 3.60 | 3.60 | 2.10 | 3.23 | 2.50 | 1.87 | 4.57 |  |  |  |  |
| **Total Forbs** | 4.04 | 10.70 | 7.71 | 41.54 | 46.64 | 40.83 | 40.01 | 0.53 | 5.57 | 15.84 | 25.91 | 22.35 | 38.87 | 28.54 | 21.33 | 5.49 | 12.26 | 12.79 | 19.45 | 20.18 | 19.80 | 19.33 | 19.51 |
| Athyrium filix-femina |  | 3.80 | 2.20 | 8.50 |  |  |  |  |  |  | 9.75 | 1.60 |  |  |  |  | 7.40 | 9.28 | 3.10 |  |  |  |  |
| Fern rhizome | 4.00 | 3.00 | 3.30 |  |  |  |  |  |  |  |  |  |  |  |  |  |  |  |  |  |  |  |  |
| Fern |  | 3.00 |  |  | 4.50 | 1.40 | 3.10 | 7.40 | 3.77 | 7.60 | 2.45 | 3.10 | 5.30 |  | 1.10 |  |  | 2.90 | 1.55 | 1.50 |  |  |  |
| Total Ferns | 0.57 | 2.90 | 0.79 | 1.70 | 0.64 | 0.20 | 0.44 | 0.62 | 1.03 | 1.38 | 2.44 | 0.43 | 0.59 |  | 0.12 |  | 1.23 | 4.35 | 0.85 | 0.19 |  |  |  |
| **Grasses** | 29.70 | 31.03 | 23.54 | 4.18 | 5.03 | 3.41 | 6.84 | 19.33 | 33.33 | 13.23 | 4.36 | 2.74 | 4.17 | 4.21 | 5.33 | 17.22 | 8.05 | 7.87 | 3.81 | 2.56 | 2.71 | 2.10 | 0.94 |
| Carex spp | 4.00 |  |  |  | 3.60 | 4.40 | 10.30 | 13.00 |  | 4.10 | 5.65 | 7.65 |  | 13.95 | 13.70 |  | 6.80 | 11.70 |  | 3.30 |  |  |  |
| Juncus |  |  |  |  |  |  |  |  |  |  |  |  |  |  |  |  |  |  | 5.10 |  |  |  |  |
| Eriophorum |  |  |  |  |  |  |  |  |  |  |  |  |  |  |  | 5.60 |  |  |  |  |  |  |  |
| Sedge Rush above | 1.30 | 2.40 |  | 2.60 |  | 2.60 |  | 2.30 |  |  | 2.90 |  |  | 1.60 | 1.80 |  |  | 2.90 | 2.90 |  |  |  | 2.60 |
| Total Sedge Rushes | 0.76 | 0.40 |  | 0.52 | 0.51 | 1.63 | 2.94 | 7.78 |  | 0.37 | 1.42 | 1.39 |  | 3.28 | 1.72 | 0.56 | 0.57 | 2.19 | 0.73 | 0.83 |  |  | 0.33 |
| Sphagnum moss | 9.20 | 2.80 | 4.50 | 3.50 | 15.20 | 3.80 | 1.70 | 2.30 |  | 1.50 |  |  |  | 1.20 | 3.53 |  | 3.15 | 3.50 | 5.10 | 3.83 | 8.60 |  |  |
| Moss | 2.65 | 1.60 | 3.80 |  |  |  |  | 4.32 | 2.53 | 5.30 | 2.80 | 3.10 |  |  |  | 6.34 | 1.45 | 8.20 | 2.55 | 2.77 | 7.75 | 3.90 | 4.60 |
| Total Moss | 2.07 | 0.73 | 1.83 | 1.40 | 2.17 | 0.54 | 0.24 | 2.54 | 0.69 | 0.62 | 0.28 | 0.28 |  | 0.13 | 1.18 | 3.17 | 0.77 | 0.98 | 1.39 | 2.48 | 3.75 | 0.49 | 1.15 |
| Mushroom |  | 1.32 | 0.33 |  |  | 0.54 |  |  |  |  |  |  |  |  |  | 0.23 |  |  |  |  |  |  |  |
| Lichen |  |  |  |  |  |  |  | 0.18 |  |  |  |  |  |  |  |  |  |  |  |  |  |  |  |
