# Supplementary material for: Is summer food intake a limiting factor for boreal browsers? Diet, temperature, and reproduction as drivers of consumption in female moose
Source: PLoS One. 2019 Oct 9;14(10):e0223617. doi: 10.1371/journal.pone.0223617 (PMC6785127; doi:10.1371/journal.pone.0223617)
Supplement: S3 Table — Fixed effects of mixed model regressions for repeated measures of A. shrub and forb digestibility (%), available N (g•100g-1), and ADF (g•g DM-1) of sampled forages, B. estimated daily dry matter intake when ordinal day and temperature were excluded (g•kg-0.75•d-1), and C. daily intakes of digestible N (g•kg-0.75•d-1) and digestible energy (MJ•kg-0.75•d-1) of female moose in relation to fecal concentration of N (g·100g-1) in summer (May–August; ordinal day 140–240) at the Kenai Moose Research Center, Kenai Peninsula, Alaska, USA in 2014–2016. The full model of main effects and interactions (X) was reduced by sequentially removing non-significant effects (—; P > 0.05 for χ2 statistic). (DOCX) [file pone.0223617.s003.docx]

A.

|  |  | Dependent Variable (Y) | | | |
| --- | --- | --- | --- | --- | --- |
| Parameters and main effects | Level | Shrub Digestibility | Forb Digestibility | Available N content | ADF content |
| Observations |  | 149 | 131 | 255 | 281 |
| χ^2^ [df] |  | 8.89[3] | 17.11[3] | 156.75[4] | 57.74[3] |
| *P* |  | 0.03 | 0.0007 | <0.0001 | <0.0001 |
| Intercept |  | 0.7456 | 0.8522 | 4.9108 | 0.1639 |
| Pen | 2 | base | base | base | base |
|  | 3 | **—** | **—** | **—** | **—** |
| Day | Ordinal Day | -0.0135 | -0.0006 | **—** | **—** |
|  | Ordinal Day^2^ | **—** | **—** | -0.0002 | <0.0001 |
|  | Ordinal Day^3^ | **—** | **—** | <0.0001 | **—** |
| Year | 2014 | base | base | base | base |
|  | 2015 | -0.0197 | -0.0343 | -0.1486 | 0.0028 |
|  | 2016 | -0.0367 | -0.0403 | -0.5732 | -0.0299 |

|  |  | Dependent Variable (Y) |
| --- | --- | --- |
| Parameters and main effects | Level | Dry Matter Intake |
| Observations |  | 175 |
| χ^2^ [df] |  | 119.4[4] |
| *P* |  | <0.0001 |
| Intercept |  | -1148 |
| Pen | 2 | base |
|  | 3 | **—** |
| Forb ADF |  | **—** |
| Forb available N |  | **—** |
| Browse ADF |  | 5063.2 |
| Browse Available N |  | 222.62 |
| Year | 2014 | base |
|  | 2015 | -105.72 |
|  | 2016 | 194.01 |

B.

|  |  | Dependent Variable (Y) | |
| --- | --- | --- | --- |
| Parameters and main effects | Level | Digestible Energy Intake | Digestible N Intake |
| Observations |  | 347 | 347 |
| χ^2^ [df] |  | 67.41[5] | 220.4[5] |
| *P* |  | <0.001 | <0.001 |
| Intercept |  | 21.8 | 33.7 |
| Fecal N |  | 0.621 | 0.798 |
| Day | Ordinal Day | -0.188 | -0.304 |
|  | Ordinal Day^2^ | <0.001 | <0.001 |
| Year | 2014 | base | base |
|  | 2015 | -1.613 | -1.900 |
|  | 2016 | -1.727 | -2.799 |

C.
