# Supplementary material for: Is summer food intake a limiting factor for boreal browsers? Diet, temperature, and reproduction as drivers of consumption in female moose
Source: PLoS One. 2019 Oct 9;14(10):e0223617. doi: 10.1371/journal.pone.0223617 (PMC6785127; doi:10.1371/journal.pone.0223617)
Supplement: S4 Table — We compared methods of removing outliers to limit the potential bias of censoring. We ran the package BACON in STATA to test for outliers with a p = 0.15 limit (Billor et al. 2000). The results of BACON on fecal output estimates present the number of outliers in the data prior to correcting for digestibility while the results of BACON on intake present the number of outliers after correcting for digestibility. We chose to censor data using a cutoff of 10% of body mass for intake results. Intake estimates over 10% of body mass would correspond to a gut capacity far above the general limit of 25% body mass in herbivores (Barboza et al. 2009). Censoring observations of intake >10% body mass reduced the mean intake by 54%. Censoring did not affect the distribution of samples over time even though the range of the number of observations per animal were reduced from 22–76 to 16–46. (DOCX) [file pone.0223617.s004.docx]

| Censor Method | Samples censored (n) | Samples remaining (n) (range of observations/animal) | Mean and range intake estimate of samples remaining  (g⋅kg^-0.75^⋅d^-1^) | Median and Range of sample days (OD) |
| --- | --- | --- | --- | --- |
| All estimates of Intake | 0 | 591 (22 – 76) | 489 (0 – 4881) | 192 (140 – 238) |
| Drop intake estimates >10% Body Mass | 217 | 348 (16 – 46) | 226 (0 – 475) | 197 (140 – 238) |
| BACON (p=0.15) on all intake estimates | 113 | 478 (17 – 64) | 266 (0 – 761) | 197 (140 – 238) |
| BACON (p=0.15) on all fecal output estimates | 116 | 477 (18 – 64) | 270 (0 – 1075) | 196 (140 – 238) |
