# Supplementary material for: Is summer food intake a limiting factor for boreal browsers? Diet, temperature, and reproduction as drivers of consumption in female moose
Source: PLoS One. 2019 Oct 9;14(10):e0223617. doi: 10.1371/journal.pone.0223617 (PMC6785127; doi:10.1371/journal.pone.0223617)
Supplement: S1 Method Validation — We devised a simulation model in the program STELLA (version 10.06 ISEE Systems, Lebanon NH) to examine the sensitivity of the estimation method to variation in the consumption of the marker ration and the quality of the diet. The model used published measures of food intake, food quality and digesta flow of moose (Clauss et al. 2011; Welch et al. 2015) to simulate pools of dry matter and marker in the rumen and the intestines (Fig A). The model predicted that marker concentrations in the feces would equilibrate after 5 days of dosing the marker at 15 mg·g-1 (Fig B, panel A) across a range of inputs for food intake (6400–15,400 g•d-1), and digestibility (0.93–0.54 g•g-1). Consequently, intakes estimated from marker concentrations in the model output were not significantly different from the simulated food intake averaged over 5 day intervals (Fig B, panel B). We validated Cr as the indigestible marker chromic oxide (Cr2O3) in five female moose (body mass 270–306) on ad libitum browse in winter (February–March). Each animal was given 500 g of a supplement (0.8 g•g-1 digestibility) containing 636 ppm. The marker was not detected in the feces before dosing at -4 days. Marker concentrations increased on the day after each dose and declined within 2 days before the next dose was consumed. The marker disappeared from the feces within 5 days of consuming the last marker dose, which is consistent with the simulation model above (Fig C). (DOCX) [file pone.0223617.s005.docx]

S1 Method Validation

| 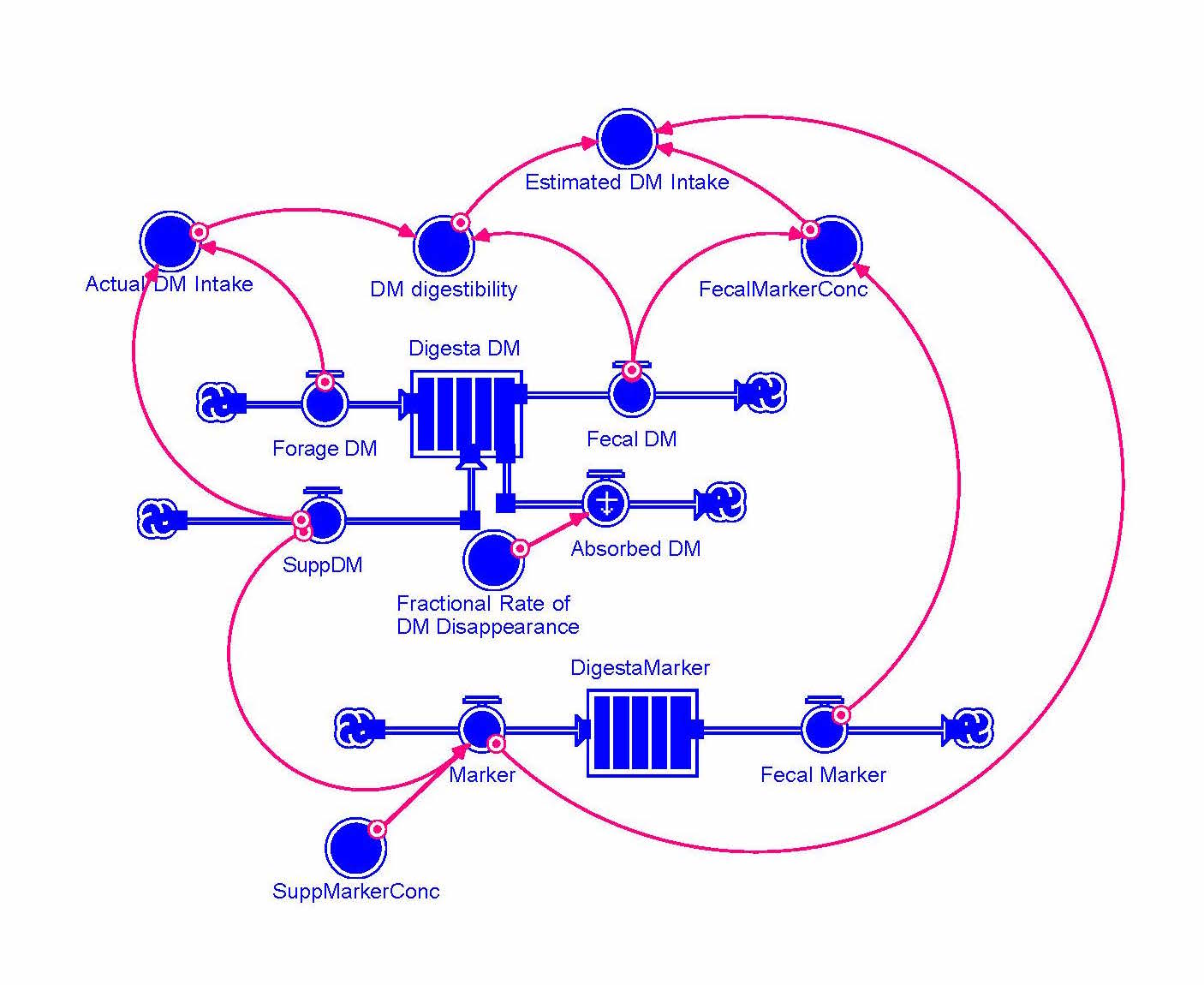 |
| --- |
| Fig A. Map of a simulation model to estimate food intake of a female moose from the excretion of a dietary marker in the feces. Boxes with vertical bars represent serial pools of dry matter and marker in the digestive tract. |

| A. |  | B. |  |
| --- | --- | --- | --- |
| Fig B. Predictions of the simulation model using fixed and variable inputs for food intake (6400 – 15,400 g•d^-1^), and digestibility (0.93 – 0.54 g•g^-1^). A. Fecal marker concentration (mg•g^-1^) after the commencement of daily doses of fixed and variable amounts of supplement (400-500g) containing 15 mg Cr•g^-1^. The equilibrated concentration of marker in the feces after day 5 increased with the digestibility of the diet and decreased with the food intake of the animal. B. Relationship between food intake (input for the model) and the food intake (g•d^-1^) estimated from the equilibrated marker concentration in the feces after 5 days of dosing. | | | |

|  |
| --- |
| Fig C. Concentration of chromium (Cr) in the feces of five female moose (body mass 270 – 306) on ad libitum browse in winter (February – March). Solid vertical lines indicate days on which each animal consumed 500 g of a supplement (0.8 g•g^-1^ digestibility) containing 636 ppm Cr as the indigestible marker chromic oxide (Cr_2_O_3_). Broken lines indicate marker concentrations of individual moose. Marker was not detected in the feces before dosing at -4 days. Marker concentrations increased on the day after each dose and declined within 2 days before the next dose was consumed. The marker disappeared from the feces within 5 days of consuming the last marker dose, which is consistent with the simulation model above. |
